# Impact of Gut Microbiota Composition on Onset and Progression of Chronic Non-Communicable Diseases

**DOI:** 10.3390/nu11051073

**Published:** 2019-05-14

**Authors:** Annalisa Noce, Giulia Marrone, Francesca Di Daniele, Eleonora Ottaviani, Georgia Wilson Jones, Roberta Bernini, Annalisa Romani, Valentina Rovella

**Affiliations:** 1 UOC of Internal Medicine-Center of Hypertension and Nephrology Unit, Department of Systems Medicine, University of Rome, via Montpellier 1, 00133 Rome, Italy; giul.marr@gmail.com (G.M.); francesca.didaniele@gmail.com (F.D.D.); e.ottaviani@hotmail.it (E.O.); georgia.wilson.jones@gmail.com (G.W.J.); valerovix@yahoo.it (V.R.); 2 PhD School of Applied Medical- Surgical Sciences, University of Rome Tor Vergata, Via Montpellier 1, 00133 Rome, Italy; 3 Department of Agriculture and Forest Sciences (DAFNE), University of Tuscia, 01100 Viterbo, Italy; roberta.bernini@unitus.it; 4 PHYTOLAB-DISIA-Department of Informatics, Statistics and Applications G. Parenti, University of Florence, Viale Morgagni, 59-50134 Florence, Italy and QuMAP-PIN-Piazza Giovanni Ciardi, 25, 59100 Prato (PO), Italy; annalisa.romani@unifi.it

**Keywords:** chronic non-communicable diseases, gut microbiota, prebiotics, probiotics, synbiotics, dysbiosis

## Abstract

In recent years, mounting scientific evidence has emerged regarding the evaluation of the putative correlation between the gut microbiota composition and the presence of chronic non-communicable diseases (NCDs), such as diabetes mellitus, chronic kidney disease, and arterial hypertension. The aim of this narrative review is to examine the current literature with respect to the relationship between intestinal dysbiosis and the insurgence/progression of chronic NCDs, analyzing the physiopathological mechanisms that can induce microbiota modification in the course of these pathologies, and the possible effect induced by microbiota alteration upon disease onset. Therapy based on probiotics, prebiotics, synbiotics, postbiotics, and fecal microbiota transplant can represent a useful therapeutic tool, as has been highlighted on animal studies. To this moment, clinical studies that intended to demonstrate the beneficial effect induced by this kind of oral supplementation on the gut microbiota composition, and subsequent amelioration of signs and symptoms of chronic NCDs have been conducted on limited sample populations for a limited follow-up period. Therefore, to fully evaluate the therapeutic value of this kind of intervention, it would be ideal to design ample population; randomized clinical trials with a lengthy follow up period.

## 1. Introduction

The human microbiota is a complex collection of microorganisms that colonizes the human body at the cutaneous, oral, respiratory, gastrointestinal, and genitourinary tract. The estimated weight of the microbiota is about 1.5 kg, and it mainly includes bacteria, but also viruses, fungi, protozoa, and archaea. In the gut, there are about one trillion bacteria (a value ten times higher respect to the number of human cells), comprising from 500 to 1000 species [1].

Even if historically the ratio between bacterial and human (B/H) cells was hypothesized to be 10:1, this has been recently criticized [2]. A study has proposed that in reality the B/H ratio is of 1:1. Such data has been deduced from the re-evaluation of the number of cells and bacteria in the human body by using new DNA analysis techniques, magnetic resonance imaging (MRI), to correctly estimate the weight of each organ, and through the revision of scientific literature pertinent to this subject [3].

The “microbiome”, meaning the genome of the microbiota combined with its environmental interactions [4], includes more than 3 million genes and is 150 times the size of the human genome. The complexity and interest in the microbiota led to the foundation in 2008 of the “MHP—Microbiome Human Project”, which was aimed to identify, characterize, and classify the microorganisms of the human microbiota [5].

The human microbiota plays a key role in the immune system maturation, metabolism of lipids, glucose, and bile acids, and in the defense against pathogens through the competition for space and nutrients, by activating the host immune system and by “priming” immune cells [6].

**Figure 1:**
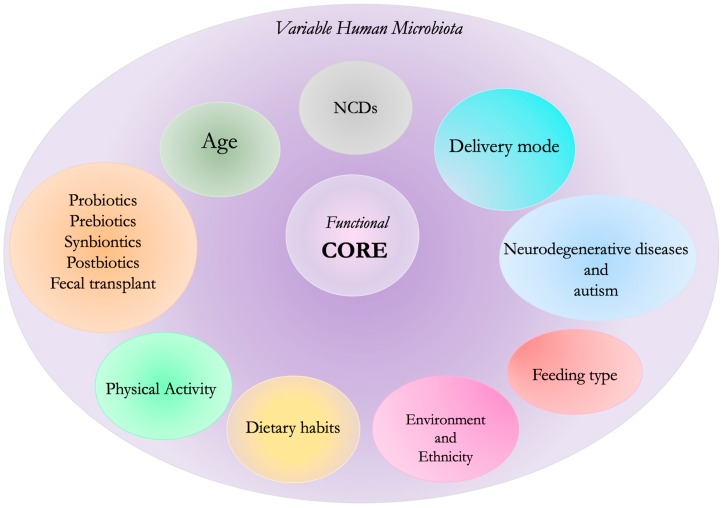
Mechanisms influencing the composition of the gut microbiota. NCDs, chronic non-communicable diseases.

This extended microbial community can be considered as a highly dynamic organ, which is sensitive to environmental insults and modifies its composition over the host’s lifespan. Its structure and activity are influenced by multiple factors that make it a unique system, which varies from individual to individual, Figure 1 [7].

In the realm of the microbiota a “core” has been identified, represented by a series of taxa constantly present in healthy subjects and absent in pathologic subjects, suggesting that the absence of such taxa may characterize dysbiosis [8]. However, successive studies have reconsidered this hypothesis because of a certain degree of taxonomic variation between heathy individuals [8,9].

A new hypothesis has identified a “healthy functional core”, which can be described as a complex of metabolic and molecular functions carried out by the microbiota but not necessarily linked to the same microorganisms in different individuals [10].

The mode of delivery influences the neonatal composition of the gut microbiota. Neonates born vaginally have microbes similar to those located in the vaginal maternal microbiota. Whilst those delivered by caesarean section have a typically cutaneous microbiota, consisting mainly of *Staphylococcus* and *Propionibacterium* spp. [11] and microbes from the hospital environment [12].

In infants, particularly during the first year of life, delivery mode has been hypothesized to affect immunological functions and gut microbiota composition. Newborns delivered by caesarean section have a reduced number of bacterial cells counts in fecal samples and a large number of antibody-secreting cells [13]. Feeding type modality in infants is an ulterior factor in microbiota modulation. Some studies have shown there is a difference in the gut microbiota composition between breast fed infants and formula fed infants [14,15]. The latter, present altered bacterial abundance particularly skewed towards the family of the Peptostreptococcaceae that contains *Clostridium difficile*, which is commonly associated with gastrointestinal and autoimmune pathologies.

Numerous studies have shown how breast-feeding is correlated to a reduced risk of developing chronic degenerative pathologies such as diabetes mellitus (DM) and obesity, and chronic inflammatory intestinal pathologies. Moreover, breast-feeding confers protection towards respiratory and gastrointestinal infections, and allergies [16,17].

*Dietary habits*, which are closely related to geographical factors, ethnicity, and food culture represent an important element for the constitution and function of the gut microbiota [18]. With regards to ethnicity, a study by Deschasaux et al. [19] conducted on 2084 healthy subjects, all resident in Amsterdam but belonging to six distinct ethnic groups, has shown that even if all the subjects lived in the same city, they still presented varying gut microbiota composition, which could be attributed to their different ethnicities. Therefore, the observed differences, characterized by varied alpha-diversity and interindividual variations, are independent of metabolic health and are partly explained by characteristics linked to ethnicity such as alimentary habits, life style, and socio-demographic factors [20].

A study by De Filippo et al. [21] compared the fecal microbiota of European Union (EU) children to that of children from the rural African village of Burkina Faso (BF). The BF children diet is characterized by high intake of fiber. BF children showed a significant increase in Bacteroidetes and reduction in Firmicutes, with an exclusive bacterial richness from the genus Prevotella and Xylanibacter, which can hydrolyze cellulose and xylan, completely absent in EU children. This suggests that the gut microbiota is influenced by polysaccharide-rich dietary content of BF children, allowing them to maximize energy intake from fiber-based diet [21].

Bacterial metabolites differ from those generated by enzymatic processes in humans, as bacterial reactions occur under anaerobic conditions and are constituted mainly by reductive or hydrolysis reactions. Studies conducted on the metabolism of polyphenols in the gut microbiota have allowed the understanding of functions performed by these compounds, and their possible health effects in humans [22,23,24]. Nowadays, the literature has highlighted the possible metabolic pathways of polyphenols by intestinal bacteria and examined their diet related metabolism [25]. In the pathophysiological mechanism of many pathological conditions, the alteration of the gut microbiota plays an essential role. In fact, the balance between the host’s immune system and the gut microbiota composition is fundamental in the maintenance of a healthy status [26].

Amongst foods able to influence gut microbiota composition, polyphenols deriving from red wine have particular importance [27,28]. Whereby, their daily consumption for only four weeks significantly enhanced the number of Enterococcus, Prevotella, Bacteroides, and Bifidobacterium genera and *Bacteroides uniformis, Eggerthella lenta, Blautia coccoides*, and *Eubacterium rectale* species [22].

Recent studies have also shown that resveratrol can positively modulate gut microbiota composition, ameliorating glucose tolerance in a murine model of obesity [28,29,30].

It is therefore, deduced that diet plays a fundamental role in modulating the composition of the gut microbiota, becoming an active part in some disease pathogenesis.

Although the association between the onset of metabolic pathologies and the alteration of the Firmicutes to Bacteroides (F/B) ratio relationship remains uncertain, recent studies have highlighted a correlation between the presence of Akkermansia and Lactobacillus genera with central obesity and fasting hyperglycemia [31,32].

Polyphenols, oligo-, and polysaccharides seem to be able to favor the growth of beneficial bacteria and inhibit that of pathogenic species [22,33,34].

The health effects of polyphenols, depends on their bioavailability. Amongst polyphenols, minor polar compounds from extra virgin olive oil, in particular hydroxytyrosol (HT), play a pivotal role in modulating the gut microbiota composition [35]. Since the concentration of HT in the body is reduced, it is hypothesized that HT may have direct effects on the gastrointestinal system, before its absorption. Therefore, the bioavailability and the beneficial effects of polyphenols on the host are related to their transformation by specific pathways via esterase, glucosidase, demethylation, and decarboxylation activities in gut microbiota [36].

Age is another factor that influences the composition of the human microbiota. At birth, the variability of the microbiota is lower because the diet is solely comprised of the mother’s milk. With time and the introduction of an ample variety of foods, the human microbiota adapts by varying and increasing its bacterial composition in order to metabolize as many foods as possible [37].

Literature evidence shows that a variety of age-related conditions such as physical frailty, and pathologies such as *C. difficile* colitis, vulvovaginal atrophy, colorectal carcinoma, and cardiovascular (CV) disease can be linked to microbiota alterations. As a future prospect, microbiota manipulation in elders could be an innovative therapeutic strategy to counteract the evolution/progression of age-related comorbidities [38].

The effect of antibiotics on the human microbiota composition is the most studied drug type interaction. Antibiotic therapies are not only effective against pathogenic microorganisms but also against the host associated microbial communities in the gut, and act by reducing the variability of the intestinal microbiota. Löfmark et al. [39] showed that even short-term antibiotic administration (one week of clindamycin) could cause long-term alterations in the commensal microbiota of healthy subjects, detectable up to two years after antibiotic administration.

Physical activity is another important factor that influences the composition and the function of the gut microbiota, by having a beneficial impact on it. A study by Clarke et al. [40], conducted on professional rugby players, demonstrated that physical exercise increases the alpha-diversity (expression of the number of species present in relation to their relative abundance and correlated to the health status of the subject) of gut microorganisms, which is significantly correlated with creatine kinase (CK) plasmatic levels and protein intake. This study strengthens the hypothesis that physical activity has a positive influence on the microbiota composition, by having an impact on its alpha-diversity [5,41]. In the same study, the authors demonstrated that athletes with lower body mass index (BMI) had significantly higher abundance of the species *Akkermansia muciniphila*. The latter is a gram-negative bacterium, which is able to degrade mucin and its presence is inversely correlated with obesity and metabolic disorders in mice and humans [42,43]. *A. muciniphila* carries out a beneficial function on the human organism because it is involved in increasing the thickness of the intestinal mucosa, bettering its tropism and protective function against pathogens [44].

Moreover, by degrading mucin it provides energetic substrates to other commensal species present in the gastrointestinal lumen [45].

The gut microbiota composition is usually characterized by bacterial members of the Bacteroidetes and Firmicutes phyla [46]. However, an important range of variation in the taxa present in the gut and interindividual variability in microbial composition has been observed, it has been supposed that the gut microbiota of most individuals can belong to one of three possible variants or “enterotypes” based on the dominant genera (Bacteroides, Prevotella, or Ruminococcus) [47]. These variants may in fact be more appropriately characterized as a ratio of the relative abundance of Bacteroides and Prevotella, with the Ruminococcus enterotype comprised into the Bacteroides group [48].

In reality, from latest studies, it has emerged that the most correct term to describe the gut microbiota composition is not via the characterization of the dominant enterotype, but rather via the evaluation of the concentration gradient of different microbial species along the gastro-intestinal tract [49,50].

Quantitative and qualitative alteration of the gut microbiota composition is termed “dysbiosis”. In recent years the correlation between altered microbiome and pathologies, including different systems of the human organism, ranging from the gastrointestinal tract to the genitourinary tract up to the central nervous system, have received increasing interest [51].

Dysbiosis has been associated with the onset of many inflammatory, non-inflammatory, and infectious diseases such as the inflammatory bowel disease, the metabolic syndrome, cancer, autoimmune conditions, and *C. difficile* infection [52].

In this review, we analyze the current literature on the correlation between the composition of human microbiota and the onset and progression of chronic non-communicable diseases (NCDs), specifically: chronic kidney disease (CKD), DM, and arterial hypertension (HTN), evaluating both animal and human studies. Biomedical studies are largely based on murine models, since the anatomy and the physiology are quite similar to that of humans. This is particularly true for the gastrointestinal apparatus; however, it must be noted that there are also some differences correlated to different diet, body composition and metabolic requirements [53].

Regarding microbiota composition, the two major phyla are common to both humans and rats (Bacteroidetes and Firmicutes) [54,55,56].

However, on a more detailed analysis of the taxonomic classification of the gut microbiota, about 85% of bacteria genera present in the rat cannot be found in humans [50].

Murine models appear as useful tool to comprehend mechanisms that correlate microbiota related pathologies, and allow scientist to perform research that would not conductible in humans, even if the results obtained are not always translatable because of species specific variations.

## 2. Methods

Current literature covering the impact of gut microbiota on onset of NCDs is analyzed and contextualized in this review. Specifically, research has been conducted on Medline (Pubmed) and Scopus. Such research examines studies published until January 2019 utilizing the words: “microbiota”, “gut microbiota” alone or in combination with “kidney”, “CKD”, “diabetes”, “hypertension”, “prebiotic”, “probiotic”, “synbiotic”, “postbiotic”, “fecal transplant”.

## 3. Gut Microbiota and Chronic Kidney Disease

Already in the 60s, a study conducted by Einheber et al. [57] demonstrated how the survival of germ-free nephrectomized mice was superior to conventionally raised nephrectomized mice. This pioneering study paved the way for further studies on the microbiota-kidney axis. Amongst the most recent studies conducted on humans one of particular interest Aranov et al. [58], which showed how colectomized hemodialysis (HD) patients have lower plasma levels of uremic toxins compared to HD patients with an intact colon, these data suggest the close relationship between the production of uremic toxins and gut microbiota.

Over the years, several studies have been conducted to characterize the gut microbiota. The first studies [5] were based on classic microbiological culturing techniques, whilst, recently, advances have been made thanks to the sequencing of either total DNA or 16S ribosomal RNA-genes. Uremia is a condition able to alter the gut microbiota composition, leading to dysbiosis. In CKD, there is a subversion of the normal intestinal balance resulting in a prevalence of Enterobacteriaceae *(Escherichia* spp., *Enterobacter* spp., *Klebsiella* spp., *Proteus* spp.), Lachnospiraceae, and Ruminococcaceae, and a reduction of Bifidobacteriaceae *(Bifidobacterium* spp.), Lactobacillaceae (*Lactobacillus* spp.), Bacteroidaceae, and Prevotellaceae [59]. In CKD there is an increase in bacteria with proteolytic activity, responsible for the production of uremic toxins (such as indoles, phenols, and trimethylamine), amines with biological activity (such as histamine, tyramine) and hydrogen compounds (such as hydrogen sulfide, methane). Additionally, there is a decrease in bacteria with saccharolytic activity responsible for the production of short chain fatty acids (SCFAs), water, CO_2_, and alcohols [60].

SCFAs introduced in the organism mainly through a fiber rich diet, perform various functions involved in the maintenance of eubiosis (a condition of equilibrium, characterized by a positive gut microbiota status) including tropism, being the main source of energy for the colonocytes, and the modulation of the gut immune system [61]. Moreover, SCFAs regulate the immunomodulatory activities of intestinal macrophages, the most abundant immune cell type in the lamina propria.

Previous studies [62] demonstrated how patients affected by intestinal chronic inflammatory diseases cause a reduction of bacteria able to produce SCFAs. The oral administration of SCAFs (n-butyrate) in rats highlighted their modulatory action in the immune response of macrophages in the lamina propria. Through the inhibition of histone deacetylase, an enzyme involved in the production of inflammatory cytokines such interleukin (IL) 6, and IL-12 suggests that the reduction of saccharolytic bacteria, and consequently of SCFAs, could exacerbate the chronic inflammatory status present in patients with CKD [63,64].

Amongst the different uremic toxins produced by proteolytic bacteria in dysbiosis of CKD patients, the most studied are: trimethylamine-N-oxide (TMAO), indoxyl sulfate (IS), and p-cresyl sulphate (PCS). TMAO derives from the metabolism of choline and carnitine in the intestinal tract and the following hepatic oxidation. Its increment is inversely correlated to a reduction of estimated glomerular filtration rate (e-GFR) [65,66,67]. Studies conducted on animal models and on humans [68,69] have demonstrated that TMAO increases CV risk and promotes atherosclerosis. However, the specific mechanism of action in which TMAO induces atherosclerosis has yet to be elucidated. It is hypothesized that TMAO acts as a uremic toxin deriving from the intestine, which contributes to the chronic inflammatory state typical of chronic renal disease. In patients with moderate-severe CKD, TMAO represents an independent predictor of CV mortality [68,69], and its levels are directly proportional to inflammatory biomarkers (such as high-sensitivity C-reactive protein (hs-CRP) and IL-6). Moreover, it is worth highlighting that TMAO levels tend to reduce and normalize [67] in patients who have undergone a renal transplant.

Sun et al. [70] observed on murine models that TMAO appears to be responsible for kidney interstitial tubular fibrosis and renal dysfunction. Moreover, enhanced TMAO levels are present in the high-fat diet (HFD) induced obese mouse model. The authors also highlighted that in the HFD mouse, renal interstitial fibrosis, and phosphorylation of SMAD3 (mothers against decapentaplegic homolog 3, acts as a regulator of cell proliferation and differentiation, its phosphorylation plays a key role in renal fibrosis) are significantly increased with respects to the values observed in mice fed a low-fat diet (LFD). SMAD3 is a member of the SMAD protein family. In HFD mouse, the authors also observed a significant increase in the levels of biomarkers of kidney dysfunction, oxidative stress, and proinflammatory cytokines compared to LFD mice, specifically: kidney injury molecule-1 (KIM-1) and plasma cystatin C (CysC), nicotinamide adenine dinucleotide phosphate (NADPH) oxidase-4, tumor necrosis factor-α (TNF-α), and IL-1 β. The confirmation of TMAO’s detrimental role in renal pathology was proven by concomitant treatment with the trimethylamine inhibitor 3,3-dimethyl-1-butanol (DMB). DMB reduced TMAO plasma concentration, and prevented the morphological and molecular changes, which were highlighted in HFD mice.

IS derives from the metabolism of tryptophan by bacterial tryptophanase and subsequent conjugation with a sulfate group in the liver. This uremic toxin may be related to oxidative stress elicitation and to the production of pro-inflammatory cytokines [71], which in turn lead to the creation of reactive oxygen species (ROS), that play a key role in the progression of CKD and in the onset of its complications. The administration of IS in uremic rats showed that the overload of this protein metabolite on residual nephrons is involved in the enhancement of bioactivity of transforming growth factor (TGF) β-1 in the kidneys. In turn, the increase of TGF β-1 increases the renal expression of metallopeptidase inhibitor 1 (TIMP1) and pro-alpha 1 collagen, leading to the worsening of glomerular sclerosis and the decline of renal function [72]. IS can be considered as a powerful predictor of overall mortality, and CV events [73], having an indirect relationship with renal function and a direct relationship with aortic calcification and pulse wave velocity (a marker of arterial stiffness) in CKD patients.

PCS derives from intestinal proteolytic fermentation of tyrosine and phenylalanine, and from the following conjugation with a sulfate group in the liver. Administration of PCS caused significant renal tubular damage in 3/4 nephrectomized rats, by enhancing oxidative stress [74]. The renal toxicity of PCS seems to be caused by its intracellular accumulation, leading to both amplified NADPH oxidase activity and ROS production, which, in turn, triggers the production of inflammatory cytokines and TGFβ-1 secretion involved in renal fibrosis. This mechanism is similar to that for the renal toxicity of IS. Both PCS and IS plasma levels are related to an increased risk of CV events and death in HD patients [75].

Mounting evidence from these studies [76] shows that dysbiotic gut microbiota, characterized by uremic toxins (such as TMAO, IS, and PCS) produced by proteolytic bacteria, should be considered one of the pathogenic factors associated with a greater risk of kidney disease progression.

Some food components may have a direct toxic action, for example, phosphates and oxalates become toxic when they accumulate during CKD. While other nutrients can be processed by the gut microbiota, directly producing uremic toxins or their precursors that are, in turn, metabolized into toxins. Higher intake of these nutrients may change the gut microbiota composition, increasing the number of bacteria that process them and causing uremic toxin production [76].

Circulating levels of nutrient-derived uremic toxins are related to an increased risk of CKD progression. These findings pave the way for future therapeutic approaches, particularly through the modification of the intestinal gut microbiota composition, in order to impact directly on the production of uremic toxins [77].

## 4. Microbiota and Diabetes Mellitus

DM is a metabolic disorder characterized by chronic hyperglycemia, which causes organ damage in the retina, kidney, nervous system and CV system [78]. Diabetic patients are in a state of low-grade chronic inflammation [79,80,81]. Over the years, evidence has been collected on the involvement of dysbiosis in the occurrence of insulin resistance and in the development of a chronic inflammatory process.

An interesting study [82] relates the alteration and increment in bacterial lipopolysaccharide (LPS) plasma concentration with dietary modification. Normally LPS plasma levels vary in a consistent manner with the circadian cycle, however, it was shown that the administration of an HFD could cause a disruptive effect on this relationship. After only four weeks, an increase in circulating LPS was demonstrated. The authors have termed this condition “metabolic endotoxemia” and have observed that it is involved in driving the expression of inflammatory cytokines like TNF-α, IL-1, and IL-6. Moreover, the role of LPS and metabolic endotoxemia in insulin resistance has been explored by inducing a suppressive mutation of CD14, an important LPS receptor. The CD14 suppressed mice showed hypersensitivity to insulin, leading to the inference that the interaction between LPS and its receptor regulates insulin sensitivity.

It has been; therefore, hypothesized that metabolic endotoxemia regulates both the glyco-metabolic state and chronic inflammation [83]. Consequently, reducing plasma LPS concentration could be a potential therapeutic weapon for the glyco-metabolic control in the DM patients [79].

Combined administration of norfloxacin and ampicillin in *ob/ob* mice (genetically modified obese mouse model, lacking the satiety hormone “leptin”) [84], greatly reduced the number of anaerobic and aerobic bacteria in the cecum. After two weeks of antibiotic combination, the authors observed a reduction in fasting glycemia and oral glucose tolerance of *ob/ob* diet-induced obese and insulin-resistant mice. Moreover, a decrease of liver triglycerides (TG), plasma LPS and an increase adiponectin was observed [85]. Therefore, the modulation of the microbiota through antibiotics could ameliorate glucose tolerance and the inflammatory process.

Interestingly, some bacteria can have a protective role against the onset of metabolic diseases. For example, the abundance of *A. muciniphila* is decreased in obese and type 2 diabetic mice, and is normalized by prebiotic feeding with oligofructose [86].

*A. muciniphila* treatment reverted HFD induced metabolic disorders, as well as fat-mass gain, metabolic endotoxemia (lowering serum LPS levels and mRNA expression of *CD11c*, biomarker of adipose tissue differentiation), adipose tissue inflammation, and insulin resistance in diet-induced obese mice. In addition, *A. muciniphila* administration re-established gut barrier function and enhanced gut endocannabinoid content (acylglycerols), which control inflammation and glucose homeostasis [43].

As demonstrated in animals, LPS levels are increased in humans affected by type 2 diabetes mellitus (T2DM) and obesity. LPS activates toll-like receptors (TLRs), which in turn induce inflammation and enhance the production of potentially diabetogenic pro-inflammatory cytokines produced by the adipose tissue, such as IL-6 and TNF-α. Furthermore, the administration of antidiabetic therapy (rosiglitazone) is responsible for the decline of the LPS and fasting serum insulin levels in previously untreated T2DM patients [87]. An interesting study has demonstrated that levels of bacteremia are correlated with the development of T2DM; in fact, the 16S rDNA concentration (a specific bacterial marker) was higher in those subjects destined to have diabetes in comparison to those who do not have a predisposition to develop the disease [88]. 16s rDNA ribosomal sequencing is a universal, rapid and accurate technology which is useful in order to identify bacteria present in examined samples. Particularly, it is advantageous in cases of uncultivable, slow growing, rare, or unusual bacteria. It may constitute a diagnostic technique in case of infections with a negative culture [89]. Zhang et al. [90] sequenced the microbial 16S rRNA genes of fecal samples of three different subgroups divided according to their glucose intolerance status (normal tolerance, prediabetes and T2DM individuals). This study showed that every progressive stage of the development of diabetes is related to a specific change in the composition of the gut microbiota. Glucose tolerance decreases along with the presence of butyrate producing bacteria such as *A. muciniphila* and *Fecalibacterium prausnitzii* and is also associated with a decrease of Verrucomicrobiae. The abundance of Streptococcus continued to decrease together with the glucose tolerance increase. These results suggest that the composition of gut microbiota could be potential marker for a high risk of DM, and the conservation of gut microbiota homeostasis in the prediabetes stage may be a strategy to delay the development of the pathology [90].

Qin et al. [91] developed a protocol termed “metagenome-wide association study” (MGWAS) based on the sequencing of the gut microbial DNA from 345 Chinese individuals, to compare the genetic content of the fecal microbiota between healthy and diabetic subjects. They identified and validated about 60,000 T2DM associated gene-sequence, showing that patients with T2DM were identifiable by a moderate grade of gut dysbiosis, more specifically by a reduction in the abundance of some butyrate-producing bacteria and a raise in opportunistic pathogens, an improvement of other microbial activities inducing sulfate reduction and oxidative stress resistance. These biomarkers could be useful for the classification of T2DM patients.

Subsequently, Karlsson et al. [92] developed a mathematical model to characterize the fecal metagenome of a cohort of European women with normal, impaired, and diabetic glucose control. Then they selected a possible metagenomic profile to accurately characterize individuals with T2DM. Interestingly, they applied their model to the previously cited Chinese cohort [91] and showed that the metagenomic markers for T2DM are different in the two cohorts examined (European and Chinese), suggesting that metagenomic predictive tools for T2DM should be defined according to age and place of origin of the individuals.

## 5. Microbiota and Arterial Hypertension

HTN is one of the most prevalent CV diseases worldwide and scientific evidence from both human and animal studies suggest that a number of factors (such as dietary habits, physical activity, pregnancy, and use of antibiotics), are closely related to HTN onset through their effect on the gut microbiota [93].

Tao Yang et al. [94] conducted a multi-step study on fecal samples containing bacterial DNA found in a spontaneously hypertensive rat (SHR) model, comparing it to that of Wistar Kyoto normotensive rats. Subsequently, they compared fecal samples from a cohort of patients with essential hypertension (EH), to a healthy subject control group. Finally, they analyzed the samples from chronic angiotensin II infusion rats (model in which hypertension is induced pharmaceutically) comparing them to the SHR. The results showed that, in the spontaneously hypertensive rats, a significant reduction in microbial abundance, diversity, and uniformity, together with an increased F/B ratio and a diminished abundance of acetate and butyrate producing bacteria were observed. The alterations observed in the SHR were comparable to the changes found in the chronic angiotensin II infusion rats, confirming that dysbiosis was independent from the mode of HTN insurgence. The EH patient cohort presented a similar dysbiotic pattern to the hypertensive rats. Successively, in order to evaluate if the eventual restoration of the F/B ratio could induce a positive effect on the blood pressure (BP), the authors administered a minocycline-based therapy to the chronic angiotensin II infusion rats. Such procedure highlighted a slight improvement of BP values as a consequence of the restored F/B.

A study by Adnan et al. [95] showed that the transplantation of SHR microbiota in normotensive rats induced an increase of systolic BP and dysbiosis by increasing the F/B ratio, confirming that the F/B ratio alteration is associated to the surfacing of systemic pathologies such as HTN.

The sequencing of 16S rRNA genes from fecal samples of Dahl salt-sensitive rats (S) [96], a genetic model of HTN, and Dahl salt-resistant rats (R), a genetic model of normotension, showed significant differences between S and R groups in gut microbiota composition. In particular, the authors observed that Bacteroidetes, particularly the S24-7 family, and the Veillonellaceae family of the Firmicutes phylum, were significantly higher in the S rats respect to the R rats. This confirms the pivotal role played by the F/B alteration in the development and maintenance of EH.

Pluznick et al. [97] reported that SCFA (specifically propionate) administration in mice produces hypotension because of the disruption of the olfactory receptor 78 (Olfr78, a receptor expressed in the juxtaglomerular afferent arteriole that mediates renin secretion in response to the production of SCFA) and the expression of G protein receptor 41 (Gpr41, a regulator of the smooth muscle cells of small resistance vessels). In addition, antibiotic therapy in the Olfr78 knockout mice reduces the gut microbiota biomass and increases BP, suggesting that SCFAs produced by the gut microbiota might modulate the BP via Olfr78 and Gpr41 activity. Therefore, accumulating evidence from these studies suggests that changes in the composition of gut microbiota plays a fundamental role in the induction and furthering the progression of HTN.

Research conducted in China [98] examined the composition and function of gut microbiota by 16s metagenomic sequencing in 196 participants, grouped in healthy control, pre-HTN (pHTN), and HTN patients. Compared to the healthy controls, the study confirmed a dramatically decreased microbial richness and diversity in both pHTN and HTN groups. Moreover, Prevotella dominated the gut enterotype of pHTN and HTN groups, which showed distinct metagenomic composition characterized by reduced bacterial species associated with a healthy phenotype, and a concomitant overgrowth of bacteria such as Klebsiella.

The microbiome characteristic in pHTN group and HTN group were similar. In addition, the fecal transplantation from hypertensive human donors to germ-free (GF) mice caused an elevation in BP, suggesting that HTN could be transferrable through the gut microbiota.

Overall, gut microbiota can contribute to the pathogenesis of HTN, and the first signs of dysbiosis can be found in the pHTN phase, suggesting that early intervention in this stage could be a future strategy to control this pathology.

Nowadays, the gut microbiota modulation appears as a useful tool in the prevention and care of dysbiosis associated with CKD, DM, and HTN. It may be modulated through the administration of antibiotics, prebiotics, and probiotics, or by fecal transplantation. The following sections will focus on the interest of probiotic, prebiotic, and synbiotic approaches in the management of these diseases.

## 6. Probiotics

The Food Agricultural Organization/World Health Organization (FAO/WHO) has defined probiotics as “live microorganisms which, when administered in adequate amounts, confer a health benefit to the host” in agreement with the International Scientific Association for Probiotics and Prebiotics (ISAPP) [99].

Probiotics are able to change the microorganism population of the gut microbiota and control the correct functioning of this ecosystem. The potential of probiotic strains has recently motivated researchers to study the production of foods with probiotic activity.

The importance of probiotic foods, such as yogurt and other fermented milks, was already recognized since the beginning of the 20th century (Table 1).

In fact, in 1907, Nobel Laureate Elie Metchnikoffn [115] suggested the value of probiotics, observing that Bulgarian peasants lived longer lives because of their yogurt consumption.

Most probiotic products today are developed with Bifidobacteria, Lactobacilli, and other lactic acid producing bacteria, such as Lactococci and Streptococci. Other probiotic strains that could induce beneficial effects on health status include the bacterial genera Bacillus, Escherichia, and Propionibacterium and some other yeast genera, mainly Saccharomyces. Probiotics are generally considered safe for human health with reduced adverse effects [116]. Several species and strains of Lactobacilli, including *Lactobacillus acidophilus*, *Lactobacillus casei*, *Lactobacillus rhamnosus*, and *Lactobacillus helveticus*, have been extensively studied in the prevention of NCDs. We are going to discuss the role of probiotics in CKD, DM, and HTN.

### 6.1. Probiotics and CKD

Several studies have been conducted in order to quantify the efficacy of probiotics. Not only in relation to their capacity to decrease the concentration of uremic toxins and to better renal function indices, but also in the role they play in order to reduce the systemic inflammatory status present in CKD patients. However, these studies have elucidated some discrepancies, which will be discussed in the following paragraph.

Wang et al. [117] showed that supplementation of probiotics containing *Bifidobacterium bifidum* A218, *Bifidobacterium catenulatum* A302, *Bifidobacterium longum* A101, and *Lactobacillus plantarum* A87 in peritoneal dialysis (PD) patients significantly reduced the serum levels of endotoxins and pro-inflammatory cytokines (TNF-α, IL-6 and IL-5), together with increasing the serum levels of anti-inflammatory cytokine (IL-10), and preserving residual renal function. It has been demonstrated that inflammatory cytokines (particularly IL-6 and TNF-α) are increased in dialysis patients and are correlated to increased CV events and overall mortality [118,119]; whilst endotoxins are both cause and markers of CKD related inflammation [120]. For such reason probiotics could be a valid instrument for improving CKD related prognosis.

The beneficial effect of probiotics on intestinal health has been known for centuries. For this reason, they have been studied in the management of end stage renal disease both in animal models and humans. In recent years, [121] these effects have been related to the progression of CKD, highlighting their anti-inflammatory, re-equilibrating action to restore eubiosis, and their antioxidant properties, reducing the ROS production by the gut microbiota.

Animal studies [122] have demonstrated that the gut microbiota can influence the concentration of uremic toxins, and the administration of probiotic “cocktails” can increase life expectancy in uremic rats, by reducing levels of urea nitrogen. Similar formulations of probiotics have been administered to CKD patients, stage III–IV, and according to the Kidney-Disease Outcomes Quality Initiative (K-DOQI) guidelines [100], they induced a statistically significant reduction in urea nitrogen levels [123].

A randomized, double-blind, placebo-controlled trial of strain-specific probiotic formulation with *Streptococcus thermophilus* (KB19), *L. acidophilus* (KB27), and *B. longum* (KB31) (Renadyl) [124] in HD patients has highlighted a series of beneficial effects elicited by Renadyl, as shown in Table 1. Most notable is Renadyl’s action on IS (also confirmed in animal models), because it has been shown how IS reduces erythropoietin (EPO) production in vitro [125,126]. Therefore, this uremic toxic has been proposed to be involved in the insurgence of anemia during CKD by inhibiting the hypoxia inducible factor (HIF), which in turn is responsible for the activation of EPO [127]. It is postulated that particular probiotic formulations (such as Renadyl) could be useful to contrast normochromic and normocytic anemia typical of CKD. This could represent a new combinatorial therapy, which would allow the reduction of weekly EPO dosage eliciting both therapeutic and pharmaco-economic advantages.

Moreover, in this study, no statistically significant changes were observed in measures of Kidney Disease Quality of Life (KDQOL) [101,124]. This data demonstrated that Renadyl administration in uremic patients is well tolerated and safe [128].

Miranda Alatriste et al. [102,129] observed that administration of substantial quantity of probiotic agents *(L. casei* shirota *8 × 10^9^ UFC* vs. *16 × 10^9^*) leads to a greater decrease (>10%) in blood urea levels in patients with stages III–IV of CKD according to K-DOQI guidelines [100]. This study demonstrates that not only the probiotic typology, but also the administered quantity influences the efficacy of the treatment. Ulterior RCTs are necessary to investigate the adequate dosage for nephropathic patients.

Miraghajani et al. [130] researched the effect of probiotic soy milk consumption on oxidative stress factors among type 2 DKD. DKD pathogenesis is conditioned by a plethora of factors, one of which is the oxido-reductive equilibrium [131]. It has been demonstrated that in T2DM patients there is a significant reduction in the plasmatic ratio between GSH/GSSH [132] and ROS hyper-production (both factors are involved in producing renal damage [133] and in the insurgence of precocious complications related to diabetes). In fact, various studies convene that increased oxidative stress in DM patients is closely correlated to the precocious insurgence of complications [103,134]. The results, as shown in Table 1, suggests that soy milk supplementation may help in the achievement of an improved antioxidant status in DKD patients, reducing risk factors correlated to its development.

A randomized, double-blind, placebo-controlled trial on HD patients investigated the effects of probiotic supplementation for a three-month period (*S. thermophilus*, *L. acidophilus*, and *B. longum*), this study highlighted discrepant result regarding the uremic profile and the gut microbiota of these patients. On one hand, there was a reduction in the fecal pH of the HD patients, a positive sign indicating an improvement in the gut microbiota flora composition. In fact, according to some studies probiotics increase bacterial production of organic acids and reduce pH in the colic lumen, thus inhibiting pathogenic growth [135,136]. On the other hand; however, rather than achieving normalization of uremic toxins, a significant increase in serum urea, potassium, and IS was observed. The trial showed that probiotic intervention did not seem to induce a positive effect on the inflammatory status and biochemical parameters in HD patients, cautioning health professionals on the administration of probiotics in these subjects.

In order to elucidate the real benefice induced by probiotics on uremic patients, given that the studies conducted to this date are all on limited samples and produced discording results, it would be necessary to carry out RCTs on a larger population and the effects should be availed by a lengthier follow-up period.

### 6.2. Probiotics and Diabetes

There is increasing evidence that oral administration of probiotics could reduce serum glucose levels and ameliorate the metabolic and inflammatory status of DM patients [104,137].

The consumption of skim milk and dahi, a traditional Indian fermented milk (containing *Lactobacillus lactis* ssp. *lactis*, *L. lactis* ssp. *cremoris*, *L. lactis* ssp. *diacetylactis*, and *Lactobacillus citrovorum*), was shown to ameliorate glycemic and lipid profile in high fructose fed (HFF) rats, as reported in Table 1 [105,106]. The authors hypothesized that some of the results could depend on the capacity of fermented to modify gut bacterial content. There is an increase in bacteria that produce SCFAs, which reduce plasmatic cholesterol. Moreover, there is an increase in bacteria involved in the de-conjugation of biliary acids. Biliary acids in this form cannot be reabsorbed by the intestinal mucosa, so their de novo synthesis increases, with consequent cholesterol consumption leading to its secondary reduction. It would be useful to test the effects of this probiotic on the lipid and glycemic profile on T2DM patients in order to assert its therapeutic potential.

A second study by same authors [107], investigated the effect of *L. acidophilus* and *L. casei* containing dahi on HFF rats. The authors propose that this kind of probiotic supplementation has a truly beneficial effect on the specimens. The study compared glycemic and lipid profiles of the HFF rats supplemented with dahi to an HFF control group after eight weeks, confirming the positive effect on such profiles, and highlighting its antioxidant effect (Table 1). In such regards, the antioxidant action of probiotics at the hepatic and pancreatic level has been proposed, as it was shown that dahi supplementation inhibited thiobarbituric acid reactive species elevation as well as decreasing reduced GSH. In conclusion, the administration of probiotics contained in dahi has induced, in animal models, multiple beneficial effects improving not only the glycemic profile, but also the lipid one and reducing oxidative stress.

A randomized controlled trial on diabetic patients [138] demonstrated that the administration of *L. casei* decreased parameters for related with glycemic metabolism (Table 1). Moreover, this study revealed a new role for *L. casei* supplementation in the glucose homeostasis. This probiotic significantly increased sirtuin1 (SIRT1) plasmatic concentration NAD-dependent deacetylase, which participates in the improvement of insulin sensitivity, its increment has a positive effect on the glycemic profile of the patients. At the same time, a decrease fetuin A (FetA) plasma concentration was observed. FetA is a serum protein, which inhibits the insulin receptor autophosphorylation and decreases efficiency of insulin signaling. The compounded increment of SIRT1 and reduction of FetA could significantly improve the glycemic status of DM patients. Therefore, the administration of this probiotics could be used as an association therapy finalized to ameliorate the glyco-metabolic status of patients.

A study by Firouzi et al. [139] demonstrated that administration of probiotics in patients with T2DM decreases urea plasma levels but does not significantly change other renal function parameters (creatinine and eGFR) and electrolytes (plasma levels of sodium, potassium), suggesting the necessity to perform further studies, regarding the role of probiotics in DM and its associated complications. It was hypothesized that uremic subjects, being characterized by dysbiosis, presented higher levels of aerobic bacteria (*Escherichia coli*) and lower levels of anaerobic bacteria (Lactobacillus and Bifidobacterium). Increased concentration of *E. coli* induced higher urea production, and an increase of intestinal pH. Therefore, the administration of Lactobacillus and Bifidobacterium could induce a pH reduction, and prevent the proliferation of aerobic bacteria [108,140].

Kijmanawat A et al. [141] showed that four weeks of probiotic supplement administration, containing *B. bifidum* and *L. acidophilus*, in women with diet-controlled gestational DM, in the late second and early third trimester, ameliorate the glycemic profile (Table 1). This evidence suggests that probiotic supplements may be considered as an adjunct treatment for glycemic control in gestational diabetes, protective for both the mother and the newborn as the intrauterine environment can influence the long-term metabolic health of the offspring.

### 6.3. Probiotics and Hypertension

The beneficial effects of probiotics on BP have been reviewed in recent years [109,142]. The literature states that some lactic acid bacteria, such as *Lactobacillus johnsonii* La1 (LJLa1), have a hypotensive action in rats [143]. A Japanese group [143] proposed that the hypotensive action of LJLa1 itself or its metabolites may be due to the regulation of the autonomic nervous system. In order to test this, LJLa1 intraduodenal injections were administered to urethane-anaesthetized rats. It was reported that these injections reduced renal sympathetic nerve activity (RSNA), reduced BP, and increased gastric vagal nerve activity (GVNA). Such study highlights how probiotics, in this case *L. johnsonii* La1, can have an antihypertensive effect through the modulation of the microbiota, reduction of pathogenic species, and a direct effect on the systems involved in regulating of arterial pressure. In recent years, the correlation between HTN and RSNA hyperactivation has received considerable attention [144]. Some experimental trials have been pursued in order to evaluate the efficacy of selective sympathectomy in HTN resistant to pharmacotherapy [110,145]. The use of probiotics; however, could represent a non-invasive, side effect free, preventative therapy.

Gómez-Guzmán et al. [146] produced a study, which claims to have demonstrated for the first time the positive effect of long-term oral supplementation of probiotics to alter the HTN profile in rats, by improving endothelial function, decreasing vascular inflammation and oxidative stress, and by reducing BP. Rats were either administered *Lactobacillus fermentum* CECT5716 (LC40) or *Lactobacillus coryniformis* CECT5711 (K8) plus *Lactobacillus gasseri* CECT5714 (LC9). Both formulae managed to restore the impaired capacity of the aortic endothelium to relax in response to acetylcholine, a vasodilator with NO-agonist activity. This probiotic treatment also acts through the restoration of eubiosis and bacterial ratio, inducing a change in the cecum microbiota, with higher counts of the *Lactobacillus* spp. cluster, and lower counts of *Bacteriodes* spp. and *Clostridium* spp. Moreover, when administered to spontaneously hypertensive rats (SHR), they decreased both NADPH oxidase activity, leading to a reduction of the aortic superoxide production, and TLR4. The latter is a factor of the innate immune system involved in system inflammation; in recent years, its putative role in the pathogenesis of HTN has emerged. Animal studies have shown how angiotensin II acts through the activation of TLR4 pathways; however, the precise mechanism has yet to be elucidated [111,147]. This study shows that probiotics represent a possible therapeutic tool for the treatment of genetic HTN.

Ahrén et al. [148] demonstrated that the supplementation of *L. plantarum* DSM 15,313 fermented blueberries significantly reduced systolic and diastolic BP, by controlling the NO dependent pathway in a hypertensive rat model treated with nitro-L-arginine methylester (L-NAME—a hypertensive drug). The beneficial effect of *L. plantarum* is enhanced by products the fermentation of blueberries, which are phenolic acids (hydrox-yphenyllactic acid, 3,4-dixydroxyphenyl-propionic acid and phenyllactic acid), which hold anti-hypertensive properties [112].

Fermented milk can be considered as functional drink acting against HTN. In recent years the capacity of probiotics to reduce BP was related to the release of bioactive peptides during the fermentation process, such as the angiotensin-converting enzyme (ACE) inhibitory peptides, having a hypotensive function similar to that of ACE-inhibitor drugs. Two tripeptides that inhibit ACE, isoleucyl–prolyl–proline (Ile-Pro-Pro) and valyl–prolyl–proline (Val-Pro-Pro), have been isolated from sour milk fermented with *L. helveticus and Saccharomyces cerevisiae* bacteria [113].

A study of Tuomilheto et al. [149] demonstrated that the administration of this product, containing the ACE inhibitory peptides in patients with mild hypertension, could have a slight BP lowering effect.

A randomized, double-blinded placebo-controlled parallel group study [114] on hypertensive patients confirmed that administration of *L. helveticus* LBK-16H fermented milk containing bioactive peptides, consumed on a daily basis, had a BP lowering effect. Particularly, systolic pressure resulted reduced by −7.6 mmHg. This information is significant, as some controlled trials have demonstrated how anti-hypertensive pharmaceuticals reduce stroke risk by 1/6 with a systolic arterial pressure reduction of 5 mmHg [150].

These two studies show how probiotics (in a similar way to pharmaceuticals) act on some of the systems involved in HTN pathogenesis. For such reason, they should be considered as a possible therapeutic tool for such pathology.

Aoyagi et al. [151] have highlighted that the risk of developing HTN is significantly lower in elderly subject who take fermented milk products containing *L. casei* strain Shirota (LcS) at least three times per week. *L. casei* owes its anti-hypertensive properties to its polysaccharide component (SG1–polysaccharide–glycopeptide complex), which increases prostaglandin I_2_ biosynthesis_,_ and reduces peripheral vascular resistance [152]. The results of Aoyagi’s study indicated that Lactobacilli could also have a protective action in the development of hypertension, as well as having a BP lowering effect.

In conclusion, probiotic intervention may be a potentially effective approach in the co-treatment of HTN through the restoration of the gut microbiota.

## 7. Prebiotics

Gibson and Roberfroid devised the prebiotic concept in 1995 [153], defining prebiotics as “a non-digestible food ingredient that beneficially affects the host by selectively stimulating the growth and/or activity of one or a limited number of bacteria in the colon, and thus improves host health”. Although this original definition has been revised several times, the main features have been maintained [154]. In 2008, FAO defined prebiotics as a “nonviable food component that confers a health benefit on the host associated with modulation of the microbiota” [155].

Prebiotics are functional food components, found naturally either in plant-based foods or from synthetic production through the enzymatic conversion of sugars. These food compounds are usually carbohydrate structures or soluble dietary fibers, which are selectively metabolized by human microbes [156].

Oligosaccharides like inulin-type fructans and galactooligosaccharides represent the best-known fibers in class of functional fibers and their prebiotic effects have been explored. In particular, it has been observed that these oligosaccharides are able to stimulate the growth of Bifidobacteria and to a lesser extent of Lactobacilli [156].

The modulation of gut microbiota via the administration of prebiotics has been studied throughout the years as a potential instrument against the development and worsening of various diseases, including CKD, DM, and HTN (Table 2).

### 7.1. Prebiotics and CKD

Prebiotics represent a valid therapeutic alternative in the modulation of the gut microbiota composition, by reducing the producing of microbial-derived uremic toxins, such as TMAO, IS, and PCS.

Most dietary approaches finalized to treat CKD and to achieve alleviation of symptoms are based on daily protein consumption restriction [157,158,170,171].

Younes et al. study the role of fermentable carbohydrate supplementation as an alternative dietary attempt, in order to lower urea plasma levels [172]. The administration of fermentable carbohydrate significantly benefits the status of hematic toxicity present in the treated subjects, as reported in Table 2. These results suggest that the use of prebiotics produces similar beneficial effects in chronic renal failure as those obtained with a low-protein diet. Moreover, such supplementation induces an increase in body weight (around 600 g in five weeks). Such result is of particular clinical relevance, as the use of fermented carbohydrates in association with a targeted dietetic-nutritional intervention, is able to reduce uremic toxin levels and contrast the insurgence of malnutrition (frequent comorbidity in nephropathic patients). Therefore, it would be advisable to perform a study on a higher number of this kind of patients in conservative therapy to confirm the efficacy of such combined intervention on the disease progression and on bodily composition.

Ramos et al. [159] investigated the effect of administering prebiotic fructooligosaccharide (FOS) on uremic toxins of CKD patients undergoing conservative therapy. The results showed a reduction of total serum and free PCS whilst no changes in urinary PCS, serum IS, and indole 3-acetic acid (IAA) were recorded. The latter is a uremic toxin responsible for endothelial dysfunction, triggering inflammation and oxidative stress, and it could be considered as an independent predictor of mortality and CV events in CKD patients. The observed clinical effect following FOS administration resulted dependent on the glomerular filtrate and the dietetic fiber/protein ratio [160]. Validating the possible therapeutic efficacy of FOS in nephropathic patients following conservative therapy.

Similar results have been found by Meijers et al. [173] in HD patients. Their study showed that prebiotic oligofructose-enriched inulin significantly reduced PCS generation rates and serum concentrations in these patients. In contrast, neither IS generation rates nor serum concentrations changed significantly. PCS appears to be a risk factor for CV pathologies; therefore, its reduction would elicit a protective effect in their insurgence. Such hypothesis is yet to be confirmed by RCTs.

A successive study by Sirich et al. [174] on HD patients has obtained only partially consistent findings Meijers’s. The enrolled HD patients received supplements containing resistant starch (RS). After six weeks of this supplementation, increasing the dietary fiber reduced the unbound, free plasma level of IS. However, the decrease of PCS levels was slighter and did not reach significance. To fully evaluate the effect elicited by probiotics on uremic toxin levels in HD patients, it would be necessary to enroll more subjects and lengthen the probiotic administration time. In case the reduction of uremic toxins was confirmed, the therapy would result useful in the detoxification of HD patients, which would be advantageous for their quality of life and life expectancy; especially because, currently, dialytic strategies are not completely effective in the removal of these toxins [161,162].

Poesen et al. [163] conducted a study in patients with eGFR between 15 and 45 mL/min/1.73 m^2^ treated with prebiotic arabinoxylan oligosaccharides (AXOS). No significant effect of AXOS on serum uremic toxins level or 24 h urinary excretion such as PCS, p-cresyl glucuronide (PCG), IS, and phenylacetylglutamine (PAG) was observed, but only a slight decrease in serum TMAO.

These studies have demonstrated how the reduction of the most notable uremic toxins of bacterial origin is only partial. Different prebiotics are able to selectively reduce uremic toxins. In conclusion, further studies are necessary to elucidate the effective therapeutic action of prebiotics in uremic patients and their role in the reduction of the gut microbiota uremic toxins.

### 7.2. Prebiotics and Diabetes

Both animal- and human-based studies have been conducted in order to comprehend the role of prebiotics in the modulation of glycometabolism in DM.

Prebiotic supplementation with oligofructose in *ob/ob* mice decreased F/B ratio, increasing Bacteroidetes prevalence [164]. This type of supplementation positively altered the gut microbiota profile, shifting it towards a status of eubiosis (increase of Bifidobacteria). In addition, prebiotics improved lipid and glycemic profiles (Table 2) and, moreover, increased the enteroendocrine L-cell number (involved in the increment of colon weight and length) and ameliorated other parameters (intestinal proglucagon mRNA expression and portal plasma glucagon-like peptide-1 level—GLP1, an incretin that increases insulin release from the pancreas). Furthermore, oligofructose prebiotic supplementation reduced adipose tissue mass, muscle lipid infiltration, oxidative stress (decreasing NADPH oxidase), and inflammation status (reducing IL-1) [165]. This new evidence highlights the potential role for prebiotics in gut microbiota modulation, and their ability to benefit the health of the subject by improving the glyco-metabolic status.

A study by Dehghan [175] has demonstrated that the daily administration of oligofructose-enriched inulin to T2DM women, significantly improved their BMI, insulin resistance, and inflammatory profile (Table 2). This confirms that prebiotics may help in the modulation of inflammatory status as well as in lowering glucose plasma levels in humans. However, this should be confirmed by ulterior studies, monitoring anti-inflammatory effects by quantifying phlogoses indexes in T2DM patients.

Since the positive role played by RS supplementation in healthy individuals and those with metabolic syndrome had already been elucidated, Bodinham et al. [166] set out to investigate its potential beneficial role in T2DM. The study was carried out on a patient sample with well-managed T2DM to whom RS was administered. The results (Table 2) suggest that the beneficial effects achieved by RS supplementation related to meal glucose handling rely on a mechanism which upregulates postprandial GLP1, without needing to modify patient lifestyle (alimentary habits and physical activity levels) and the dose and type of hypoglycemic oral medication [176].

A study by Aliasgharzadeh et al. [167] examined the effects of resistant dextrin (a soluble prebiotic fiber) on insulin resistance and inflammation in T2DM women, as reported in Table 2. Particularly notable are the modifications in two insulin related distinct indexes, namely a decrease in HOMA (homeostasis model assessment of insulin resistance, a computer-solved model used to quantify insulin resistance and beta-cell function) and an increase in QUICKI (quantitative insulin sensitivity check index, determining insulin sensitivity). These findings show that this type of dietary fiber could act as a valuable supplementation for the control of T2DM, thanks to its anti-inflammatory and insulin resistance modulatory properties. Furthermore, a reduction trend in the concentration of FBG, HbA1c and hs-CRP was observed, even though it was not significant. Consequently, it was deduced that resistant dextrin supplementation could reduce the inflammatory state and improve insulin resistance in women with T2DM [168].

### 7.3. Prebiotic and Hypertension

Marques et al. [169] found that the consumption of a high fiber diet or acetate supplementation changed the intestinal microbiota composition and incremented the abundance of acetate-producing bacteria. Both fiber and acetate decrease gut dysbiosis, measured by F/B ratio, and increase the prevalence of *Bacteroides acidifaciens*, a bacterium involved in the prevention of obesity and improvement of insulin sensitivity in mice [177]. Both high-fiber diet and acetate supplementation, as shown in Table 2, significantly decreased the BP, cardiac fibrosis, and left ventricular hypertrophy. In addition, acetate markedly reduced renal fibrosis, sign of dysfunction, and CKD. Transcriptome analysis showed that the protective role of high fiber and acetate was related to the down-regulation of cardiac and kidney early growth response protein 1 (Egr1), a nuclear protein involved in cardiac hypertrophy, cardio-renal fibrosis, and inflammation. Prebiotics can; therefore, intervene in NCDs, modulating gut microbiota composition and acting directly on cellular systems involved in the evolution of such pathologies.

Obese patients subjected to a diet based on whole grain, a traditional medicinal Chinese food, and prebiotics (WTP diet), showed decreased BP and body weight, accompanied by increased insulin sensitivity and improvement of the lipid profile (HDL, LDL, PTC, and TG) [178]. An amelioration of dysbiosis was also evident, with a reduction of endotoxin-producing opportunistic pathogens (Enterobacteriaceae and Desulfovibrionaceae) and an increase of gut barrier-protecting bacteria (Bifidobacteriaceae). Moreover, an LPS reduction was observed together with proinflammatory cytokines reduction, and anti-inflammatory adipokine increase (Table 2). In obese subjects, it may be useful to flank a hypocaloric diet with prebiotics, capable of reducing weight, BP, improving the glycemic and lipid profile, and limiting the low-grade chronic inflammatory status present in such condition.

Rault-Nania et al. [179] studied the effect of the supplementation with different inulin-type fructan fractions towards common characteristics of the metabolic syndrome in fructose-fed rats (a typical model of this syndrome). The most effective were the long-chain inulin and oligofructose-enriched inulins. Their supplementation prevented fructose induced CV and kidney damage, as reported in Table 2. All inulin-type fructan-containing diets prevented fructose-induced hypertriglyceridemia; however, their mechanism of action is yet to be elucidated.

Hsu et al. [180] conducted an interesting study regarding the effect of a maternal high fructose diet that induced HTN in the adult offspring. Maternal diet is proposed to have long-term developmental programming hypertensive effects on the adult offspring’s phenotype. The supplementation therapy, which involved either the administration of probiotic *L. casei* or prebiotic inulin, was shown to prevent HTN in the adult offspring. Therefore, this kind of intervention during gestation can be considered as a reprogramming strategy.

## 8. Synbiotics

In combination, prebiotics and probiotic bacteria create synbiotics, which can compound prebiotic and probiotic benefits (Table 3).

The term synbiotic refers to the synergy present between prebiotic substrate and probiotic organisms. The advantage of such combination lies in the increased survival of the probiotics while passing through the upper intestinal tract, improving and stimulating subsequent implantation and growth [181,182].

Eslamparast et al. [183] conducted a study on a group of subjects affected by metabolic syndrome. They were administered either with synbiotic capsules (*L. casei, L. rhamnosus, S. thermophilus, Bifidobacterium breve, L. acidophilus, B. longum*, and *Lactobacillus bulgaricus*, plus fructo-oligosaccharide) and fructo-oligosaccharide, or with placebo capsules. The subjects were instructed to follow an energy balanced diet and to perform physical activity. Results showed that the group treated with synbiotics had significantly improved fasting blood glucose and insulin resistance compared to the placebo group, signifying that synbiotics can have the important role of optimizing the effect of diet and exercise regimens in subjects affected by metabolic syndrome.

A. Ahmadi et al. [184] study determined the effects of symbiotic capsule containing *L. acidophilus*, *L. casei*, and *B. bifidum* plus inulin in subjects affected by gestational diabetes. After six weeks of daily capsule administration, serum insulin levels and HOMA function were decreased, while QUICKI was increased. In addition, the serum TG and VLDL concentrations were significantly decreased. The results indicate that synbiotic therapy could be a valuable approach in managing insulin and lipid parameters in gestational diabetes.

Pavan et al. [185] examined the effects of prebiotic and probiotic supplementation plus low protein diet in order to evaluate the effect of this combined approach on the progression of CKD. It was observed that this kind of therapeutic treatment was able to decrease the decline in eGFR in stage III–IV CKD patients, compared to the dietary restriction alone. The administration of synbiotics, associated to a low-protein diet may therefore enhance the efficacy of the diet in order to slow down CKD progression.

A study by Guida et al. [186] suggests that treatment with synbiotics (containing L. *plantarum*, L. *casei* ssp. *rhamnosus*, L. *gasseri*, *Bifiodbacterium infantis*, B. *longum*, L. *acidophilus*, *Lactobacillus salivarius*, *Lactobacillus sporogenes*, S. *thermophilus*, inulin, and tapioca-resistant starch), may be effective in lowering plasma PCS concentrations in kidney transplant recipients (KTR). A pilot study was conducted on 36 KTR and showed that after 30 days of treatment plasma PCS decreased by 30%, compared to baseline, in the synbiotic treated group, whilst no significant changes in renal function, glycemia, plasma lipids, or albumin concentration were observed. Therefore, successive studies on a larger scale and with a longer follow up period are required in order to determine if such intervention also has some cardioprotective roles.

Finally, synbiotic gel (*L. acidophilus, B. lactis* and inulin) administration for the control of gastro-intestinal symptoms (GIS) and complications in HD patients has been explored. After a two-month long treatment, there was a significant reduction in the frequency and severity of symptoms such as vomit, heartburn, and stomachache; evaluated thanks to a self-administered questionnaire. The study concluded that synbiotic gel treatment is a safe yet simple strategy to control common GIS during HD [187].

## 9. Postbiotics and Fecal Microbiota Transplant

In recent years, postbiotics (metabiotics, biogenics, or metabolites/cell-free supernatants) have emerged as a potential instrument to modulate the gut microbiota [188]. They are intracellular soluble factors secreted by live bacteria as metabolic products or released after bacterial lysis [189].

Postbiotics include enzymes (for example NADH-peroxidase, glutathione peroxidase), proteins (glutathione), polysaccharides, organic acids (e.g., propionic acid), lipids (SCFA), teichoic acids, and cell surface proteins. In the majority of cases, postbiotics are derived from *Lactobacillus* and *Bifidobacterium* strains, more seldom from other bacteria such as Streptococcus and Faecalibacterium species [190]. These soluble factors have demonstrated beneficial effects on the health status, by carrying out antimicrobial, antioxidant, and immunomodulatory functions, which positively influence the microbiota composition [191]. Currently, no studies have been conducted to elucidate the effect on NCD in humans [192]. Animal studies have been conducted, particularly in the poultry industry. These highlighted the postbiotic power to inhibit pathogens, increase the weight, and ameliorate microbial composition and inflammatory profile [193,194].

A study by Sokol et al. [195] has demonstrated, in an animal model of Crohn disease, a beneficial effect induced by the administration of *F. prausnitzii* or its supernatant on the correction of dysbiosis and reducing inflammation. A successive study by Cavallari et al. [196] conducted in a murine model of obesity-induced insulin resistance via IRF4, has demonstrated an increase in insulin sensitivity following administration of muramyl-dipeptide derived from the bacterial cell wall. The administration postbiotics derived from lactobacillus was capable to contrast inflammation induced by diseases of the small intestine or from Salmonella infection in an ex-vivo organ culture model [197].

Postbiotic use represents a valid therapeutic approach in NCDs, as it could improve the chronic inflammatory status in such pathologies and limit the growth of pathogenic species, contrasting dysbiosis.

Fecal microbiota transplantation (FMT) is an innovative treatment that is still in an experimental stage. FMT consists in the administration of a solution containing fecal matter from a healthy donor to the gastrointestinal tract of the receiving patient, and it is proposed as a useful tool for the reinstallation of eubiosis [198]. The administration may occur through various methods: nasogastric tube, nasojejunal tube, esofagastroduodenoscopy, colonoscopy, or enema [199]. To reduce the risk of transmission of infections or other pathologies, it is fundamental that the donor undergoes rigorous screening for pathologies with an infectious, neoplastic, metabolic, and autoimmune basis [200]. FMT is considered safe and well tolerated [201]; however, not completely free of side effects such as abdominal discomfort, bloating, transient fever, flatulence, constipation, vomiting, and diarrhea [202]. Few studies are related to the long-term effects of FMT, such as pathologies related to microbiota composition changes like obesity, DM, neoplasia, asthma, and autism [203]. Therefore, it is essential to perform more studies with an extensive follow up period post FMT. Currently, the most well-known pathology for which FMT is effective is severe or recurrent *C. difficile* infection [204]. Still in a preliminary phase instead, is the utilization of FMT for the treatment of metabolic, CV, autoimmune, and neurologic conditions [205]. Concerning this, is an important study conducted by Vrieze et al. [206], which demonstrates how lean donor microbiota infusion determines an increase in insulin sensitivity in metabolic syndrome sufferers. Ulterior studies are required in order to validate the efficacy of FMT on pathologies associated with dysbiosis.

## 10. Conclusions

Over the last years, the gut microbiota has received increasing interest from scientific literature, and has started to be considered as a real “new organ”, that influences numerous biological functions of the organism, such as immunity, digestion, and metabolism. Thanks to new sequencing technologies, an enormous complexity and a substantial number of potential genes of the microbiota have been identified. Available clinical and experimental evidence has established the clear role of the microbiota in chronic NCDs. Initially scientific data was uniquely generated from rodent models, recently; however, the importance of a healthy gut microbiota is being demonstrated in humans. Administration of probiotics and prebiotics has been widely used in order to manipulate the gut microbiota. However, although several studies reported encouraging results, long term efficacy of this treatment is still being researched. In fact, one of the future challenges relating to the use of probiotics and prebiotics is linked to producing standardized safety guidelines for the use of these supplements in humans (particularly in order to determine side effects such as bloating, flatulence, and generalized gastrointestinal discomfort). Therefore, additional studies and randomized controlled trials are required for a deeper understanding of the clinical impact of gut microbiota manipulation. Another interesting and intricate challenge is to understand whether the administration of these supplements may lead to genetic interactions via genetic exchange between the digested strains and the indigenous flora, area of research, which remains currently virtually unexplored [207]. For the future, large prospective cohort studies could provide more evidence and information on clinical relevance of the microbiota as a potential pathogenic factor for the development of chronic NCDs.

## Figures and Tables

**Table 1:** Probiotics effects on CKD, DM and HTN.

Author	Year	Study Population	Type of the Study	Primary Outcome	*p* Value for Primary End-Point
Natarajan R [100]	2014	22 HD patients	RDBPC	Reduction in:	
				WBC count	*p* < 0.057
				CRP serum level	*p* < 0.071
				Total IG level	*p* < 0.078
Miranda Alatriste PV [101]	2014	30 stage III–IV CKD patients	RCT	Decrease in the serum urea level	*p* = 0.031
Miraghajani M [102]	2017	48 DKD patients	RCT	Decrease in:	
				Oxidized GSH	*p* = 0.03
				Increase in:	
				GSH	*p* = 0.01
				GSH peroxidase	*p* = 0.001
				GSH reductase	*p* = 0.02
Borges NA [103]	2018	46 HD patients	RDBPC	Increase in:	
				Serum urea	*p* = 0.2
				Potassium	*p* = 0.2
				IS	*p* = 0.2
				Decrease in:	
				Fecal pH	*p* = 0.1
Yadav H [104]	2006	39 high fructose-induced T2DM fed rats	RCT	Improvement of glycemic and lipid profile (blood glucose, HbA1c, glucose intolerance, plasma insulin, liver glycogen, PTC, TG, LDL, VLDL HDL, and FFA)	*p* < 0.05
Yadav H [105]	2007	18 high fructose-induced T2DM fed rats	RCT	Improvement of glycemic and lipid profile (blood glucose, HbA1c, glucose intolerance, plasma insulin, liver glycogen, PTC, TAG, LDL, VLDL, and FFA) and oxidative stress markers (GSH, TBARS)	*p* < 0.05
Khalili L [106]	2019	40 T2DM patients	RCT	Increase in SIRT1	*p* = 0.04
				Decrease in:	
				Fet1	*p* = 0.023
				FBG	*p* = 0.013
				HbA1c	*p* = 0.07
				Insulin	*p* = 0.028
				HOMA.IR	*p* = 0.007
Firouzi S [107]	2015	136 T2DM patients	RDBPC	Decrease in the serum urea level	*p* < 0.05
Kijmanawat A [108]	2019	57 Pregnant women with diet-controlled gestational DM	RDBPC	Decrease in:	
				FBG	*p* = 0.034
				Fasting plasma insulin	*p* = 0.001
				HOMA.IR	*p* = 0.001
Tanida M [109]	2005	Urethane-anesthetized rats	RCT	Decrease in:	
				RSNA	*p* = 0.0005
				BP	*p* = 0.0005
				Increase in:	
				GVNA	*p* = 0.0005
Gomez-Guzman M [110]	2015	40 spontaneously hypertensive rats	RCT	Decrease in:	
				Systolic BP	*p* < 0.01
				Increase in the relaxation induced by acetylcholine Increase in:	
				eNOS activity	*p* < 0.05
				Phosphorylation of eNOS and cardiac and renal hypertrophy	*p* < 0.05
Ahrén IL [111]	2015	54 induced hypertensive rats	RCT	Decrease in BP	*p* < 0.05
Tuomilehto J [112]	2004	60 patients with mild HTN	RCT	Decrease in:	
				Systolic BP	*p* = 0.0668
				Diastolic BP	*p* = 0.92
Jauhiainen T [113]	2005	94 hypertensive patients	RDBPC	Decrease in:	
				Systolic BP	*p* = 0.01
				Diastolic BP	*p* = 0.48
Aoyagi Y [114]	2017	352 normotensive patients	RCT	Decrease of the risk of developing HTN	*p* = 0.037

BP, blood pressure; CKD, chronic kidney disease; CRP, *C-reactive protein*; *DKD*, *diabetic kidney disease*; eNOS, nitric oxide synthase; FBG, fasting blood glucose; FetA: fetuin A; FFA: free fatty acid; GSH, glutathione; GVNA, gastric vagal nerve activity; HbA1c, glycated hemoglobin; HD, hemodialysis; HDL: high-density lipoprotein; HOMA, homeostatic model assessment; HOMA-IR, homeostatic model assessment for insulin resistance; HTN, arterial hypertension; IG, indoxyl glucuronide; IS, indoxyl sulfate; LDL, low-density lipoprotein-cholesterol; PTC, plasma total cholesterol; RCT, randomized control trial; RDBPC, randomized, double-blind, placebo-controlled; RSNA, renal sympathetic nerve activity; SIRT1: Sirtuin1; T2DM, diabetes mellitus type 2; TAG, triacylglycerol; TBARS: thiobarbituric acid reactive species; TG, triglycerides; VLDL, very-low-density lipoprotein-cholesterol; WBC, white blood cell.

**Table 2:** Prebiotics effects on CKD, DM and HTN.

Reference	Year	Study Population	Type of the Study	Primary Outcome	*p* Value for Primary End-Point
Younes H [157]	2006	9 CKD patients	Single-blinded prospective randomized trial	Increase in:	
				Urea nitrogen excretion in stool	*p* < 0.01
				Decrease in:	
				Urinary nitrogen excretion	*p* < 0.01
				Plasma urea	*p* < 0.05
Ramos CI [158]	2018	50 non-diabetic non-dialysis-dependent CKD patients	RDBPC	Decrease in:	
				Serum total PCS	*p* = 0.07
				Serum free PCS	*p* = 0.07
Meijers BKI [159]	2010	22 HD patients	Single center, non-randomized, open-label phase I/II study	Decrease in:	
				PCS generation rates	*p* = 0.007
				PCS serum concentrations	*p* = 0.03
Sirich TM [160]	2014	56 HD patients	Single-blinded prospective randomized trial	Decrease in:	
				Serum free IS	*p* = 0.04
				Serum free PCS	ns
Poesen R [161]	2016	40 CKD patients with eGFR between 15 and 45 mL/min/1.73 m^2^	Randomized, placebo-controlled, double-blind, cross-over study	No significant decrease of serum uremic toxins level or 24h urinary excretion	ns
Everard A [162]	2011	10 *ob/ob* mice	RCT	Improvement of plasma glucose profile	*p* < 0.05
				Increase in:	
				White adipose tissue weight	*p* < 0.05
				Leptin sensitivity	*p* < 0.05
				Proglucagon mRNA expression	*p* < 0.05
				Enteroendocrine cell activity	*p* < 0.05
Dehghan P [163]	2014	52 women with T2DM	RCT	Decrease in:	
				BMI	*p* < 0.05
				FBG	*p* < 0.05
				Hb1Ac	*p* < 0.05
				IL-6	*p* < 0.05
				TNF-α	*p* < 0.05
				LPS	*p* < 0.05
Bodinham CL [164]	2014	17 T2DM patients	RCT	Decrease in:	*p* = 0.045
				Postprandial glucose concentrations	
				NEFA	*p* = 0.04
				Fasting GLP1	*p* = 0.049
				Increase in:	
				Glucose uptake across the forearm muscle	*p* = 0.077
				Postprandial GLP1 excursions	*p* = 0.009
Aliasgharzadeh A [165]	2015	75 T2DM patients	RCT	Decrease in:	*p* < 0.05
				FBI	*p* < 0.05
				HOMA-IR	*p* < 0.05
				QUICKI	*p* < 0.05
				TNF-α	*p* < 0.05
				IL-6	*p* < 0.05
				Endotoxin	*p* < 0.05
				MDA	*p* < 0.05
Marques FZ [166]	2017	64 hypertensive mice	RCT	Increase in:	
				Acetate-producing bacteria	*p* = 0.0001
				*Bacteroides acidifaciens* species bacteria	*p* = 0.0001
				Decrease in:	
				F:B ratio	*p* = 0.0001
				Systolic BP	*p* = 0.0002
				Diastolic BP	*p* = 0.0001
				Glomerular fibrosis	*p* = 0.0001
				Tubulointerstitial fibrosis	*p* = 0.008
				Cardiac perivascular and interstitial fibrosis	*p* = 0.001
				Left ventricular hypertrophy	*p* < 0.05
Xiao S [167]	2014	123 obese patients	Self-controlled clinical trial	Decrease in:	
				Bifidobacteriaceae bacteria	*p* = 0.05
				Systolic BP	*p* = 0.01
				Diastolic BP	*p* = 0.05
				Body weight	*p* = 0.01
				BMI	*p* = 0.01
				FBI	*p* = 0.01
				FBG	*p* = 0.01
				HOMA-IR	*p* = 0.01
				HbA1c	*p* = 0.01
				TG	*p* = 0.01
				PTC	*p* = 0.01
				LDL	*p* = 0.05
				TNF-α	*p* = 0.05
				IL-6	*p* = 0.01
				CRP	*p* = 0.05
				Increase in: HDL	*p* = 0.05
Rault- Nania MH [168]	2008	40 high fructose-fed rats	RCT	Prevention of:	
				BP elevation	*p* = 0.028
				Hypertriglyceridemia	*p* = 0.036
				Susceptibility to heart peroxidation	*p* = 0.0001
Hsu CN [169]	2018	8 male adult offspring born to high fructose-fed mothers	RCT	Decrease in:	
				BP	*p* < 0.05

BMI, body mass index; BP, blood pressure; CKD, chronic kidney disease; CRP, *C-reactive protein*; eGFR, estimated glomerular filtration rate; F:B ratio, Firmicutes to Bacteroides ratio; FBG, fasting blood glucose; FBI, fasting blood insulin; FFA: free fatty acid; GLP1, Glucagon-like peptide-1; HbA1c, glycated hemoglobin; HD, hemodialysis; HDL: high-density lipoprotein; HOMA-IR, homeostatic model assessment for insulin resistance; IG, indoxyl glucuronide; IL-6, interleukin-6; IS, indoxyl sulfate; LDL, low-density lipoprotein-cholesterol; LPS, lipopolysaccharide; NEFA, non-esterified fatty acid; PCS, p-Cresyl sulfate; PTC, plasma total cholesterol; QUICKI, quantitative insulin sensitivity check index; RCT, randomized control trial; RDBPC, randomized, double-blind, placebo-controlled; T2DM, diabetes mellitus type 2; TG, triglycerides; TNF-α, tumor necrosis factor-α. VLDL, very-low-density lipoprotein-cholesterol.

**Table 3:** Synbiotic effects on CKD, DM and HTN.

Reference	Year	Study Population	Type of the Study	Primary Outcome	*p* Value for Primary End-Point
Eslamparast T [179]	2014	38 patients with metabolic syndrome	RDBPC	Decrease in:	
				FBG	*p* < 0.001
				HOMA-IR	*p* < 0.001
				TG	*p* < 0.001
				PTC	*p* < 0.01
				Increase in:	
				HDL	*p* < 0.001
Ahmadi S [180]	2016	70 patients with gestational diabetes	RDBPC	Decrease in:	
				Insulin plasma level	*p* = 0.005
				TAG plasma level	*p* < 0.001
				VLDL plasma level	*p* < 0.001
				HOMA-IR	*p* = 0.003
				HOMA for β cell function	*p* = 0.008
				Increase in:	
				QUICKI	*p* = 0.02
Pavan M [181]	2016	24 patients with CKD from stage III-V not on renal replacement therapy	Randomized controlled and open-label trial	A lower declining of eGFR	*p* < 0.001
Guida B [182]	2017	36 KTRs	Single-center, parallel group, double blinded, randomized study	Decrease of PCS level	*p* < 0.05
Viramontes-Horner D [183]	2015	22 HD patients	RDBPC	Reduction in the presence and severity of gastrointestinal symptoms	*p* < 0.05

CKD, chronic kidney disease; eGFR, estimated glomerular filtration rate; FBG, fasting blood glucose; FFA: free fatty acid; HD, hemodialysis; HDL, high-density lipoprotein; HOMA, homeostatic model assessment; HOMA-IR, homeostatic model assessment for insulin resistance; KTR, *kidney* transplant recipients; PCS, p-Cresyl sulfate; PTC, plasma total cholesterol; QUICKI, quantitative insulin sensitivity check index; RDBPC, randomized, double-blind, placebo-controlled; TAG, triacylglycerol TG, triglyceride; VLDL, very-low-density lipoprotein-cholesterol.

## Abbreviation

ACE	Angiotensin-converting enzyme
AXOS	Arabinoxylan oligosaccharides
BF	Burkina Faso
BMI	Body mass index
BP	Blood pressure
CK	Creatine kinase
CKD	Chronic kidney disease
CRP	C-reactive protein
CV	Cardiovascular
CysC	Cystatin C
DKD	Diabetic kidney disease
DM	Diabetes mellitus
DMB	3,3-Dimethyl-1-butanol
e-GFR	Estimated glomerular filtration rate
Egr1	Early growth response protein 1
EH	Essential hypertension
EPO	Erythropoietin
EU	European Union
F/B	Firmicutes to Bacteroides ratio
FAO	Food Agricultural Organization
FBG	Fasting blood glucose
FBI	Fasting blood insulin
FDY	Freeze-dried powdered yacon
FetA	Fetuin A
FFAs	Free fatty acids
FMT	Fecal microbiota transplant
FOS	Fructooligosaccharides
GF	Germ-free
GLP1	Glucagon-like peptide-1
Gpr41	G protein receptor 41
GSH	Glutathione
GVNA	Gastric vagal nerve activity
HbA1c	Glycated hemoglobin
HD	Hemodialysis
HDL	High-density lipoprotein
HFD	High-fat diet
HFF	High fructose fed
HIF	Ipoxia-inducible factor
HOMA	Homeostasis model assessment of insulin resistance
hs-CRP	High-sensitivity C-ractive protein
HT	Hydroxytyrosol
HTN	Arterial hypertension
IAA	Indole 3-acetic acid
Ile-Pro-Pro	Isoleucyl–prolyl–proline
IS	Indoxyl sulfate
ISAPP	International Scientific Association for Probiotics and Prebiotics
K-DOQI	Kidney-Disease Outcomes Quality Initiative
KDQOL	Kidney Disease Quality of Life
KIM-1	Kidney injury molecule-1
KTR	Kidney transplant recipients
L-NAME	Nitro-L-arginine methylester
LcS	*Lactobacillus casei* strain Shirota
LDL	Low-density lipoprotein-cholesterol
LFD	Low-fat diet
LPS	Lipopolysaccharide
MDA	Malondialdehyde
MGWAS	Metagenome-wide association study
MHP	Microbiome Human Project
NADPH	Nicotinamide adenine dinucleotide phosphate
NCD	Chronic non-communicable diseases
NEFA	Non-esterified fatty acid
NO	Nitric oxide
Olfr78	Olfactory receptor 78
PAG	Phenylacetylglutamine
PCG	p-cresyl glucuronide
PCS	p-cresyl sulphate
PD	Peritoneal dialysis
pHTN	pre-hypertension
PTC	Plasma total cholesterol
QUICKI	Quantitative insulin sensitivity check index, determining insulin sensitivity
R	Dahl salt-resistant
RCTs	Randomized clinical trials
ROS	Reactive oxygen species
RS	Resistant starch
RSNA	Renal sympathetic nerve activity
S	Dahl salt-sensitive
SCFAs	Short chain fatty acids
SHR	Spontaneously hypertensive rats
SIRT1	Sirtuin1
SMAD3	Mothers against decapentaplegic homolog 3
TBARS	Thiobarbituric acid reactive species
TG	Triglycerides
TGF	Transforming growth factor
TIMP1	Metallopeptidase inhibitor 1
TLRs	Toll-like receptors
TMAO	Trimethylamine-N-oxide
TNF-α	Tumor necrosis factor-α
Val-Pro-Pro	Valyl–prolyl–proline
VLDL	Very-low-density lipoprotein-cholesterol
WBC	White blood cell
WHO	World Health Organization
